# Supplementary material for: An Injectable Chitosan-Based Self-Healable Hydrogel System as an Antibacterial Wound Dressing
Source: Materials (Basel). 2021 Oct 11;14(20):5956. doi: 10.3390/ma14205956 (PMC8539725; doi:10.3390/ma14205956)
Supplement: Supplementary file 1 [file materials-14-05956-s001.zip › materials-1385469-supplementary.pdf]

An injectable Chitosan-based self-healable hydrogel system as an antibacterial wound dressing

Xiaoyu Wang[†]*, Rijian Song*[†]*, Melissa Johnson, Sigen A, Zhonglei He, Cameron Milne, Xianqing Wang, Irene Lara-Sáez, Qian Xu*, and Wenxin Wang**

The Charles Institute of Dermatology, School of Medicine, University College Dublin, Dublin 4, Ireland. Eircode: D04 V1W8; xiaoyu.wang2@ucdconnect.ie (X.W.); rijian.song@ucdconnect.ie (R.S.); melissa.johnson@ucdconnect.ie (M.J.); sigen.a@ucd.ie (S.A.); zhonglei.he@ucd.ie (Z.H.); cameron.milne@ucdconnect.ie (C.M.); xianqing.wang@ucdconnect.ie (X.W.); irene.lara-saez@ucd.ie (I.L.-S.)

* Correspondence: qian.xu@ucd.ie (Q.X.); wenxin.wang@ucd.ie (W.W.)

† Co-first author

Contents

Supplementary tables 1-2

Supplementary figures 1-2

Supplementary references

Table S1. Element analysis results of CTS-CHO.

Sample	C (%)	N (%)	H (%)	OD (%)
CTS	41.77	7.12	6.73	N/A
CTS-CHO	38.88	5.32	6.09	14.75%

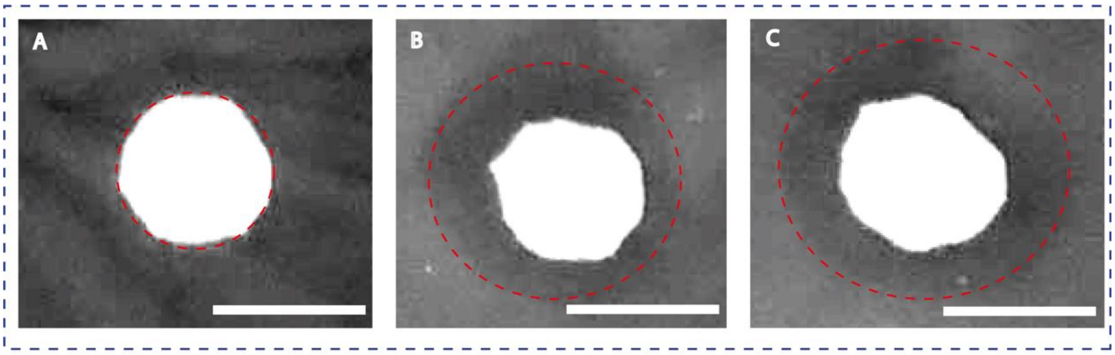

Figure S1. Images of bacterial growth inhibition profile at different concentrations: A: 10 mg/mL; B: 30 mg/mL; C: 50 mg/mL. Scale bar: 8 mm.

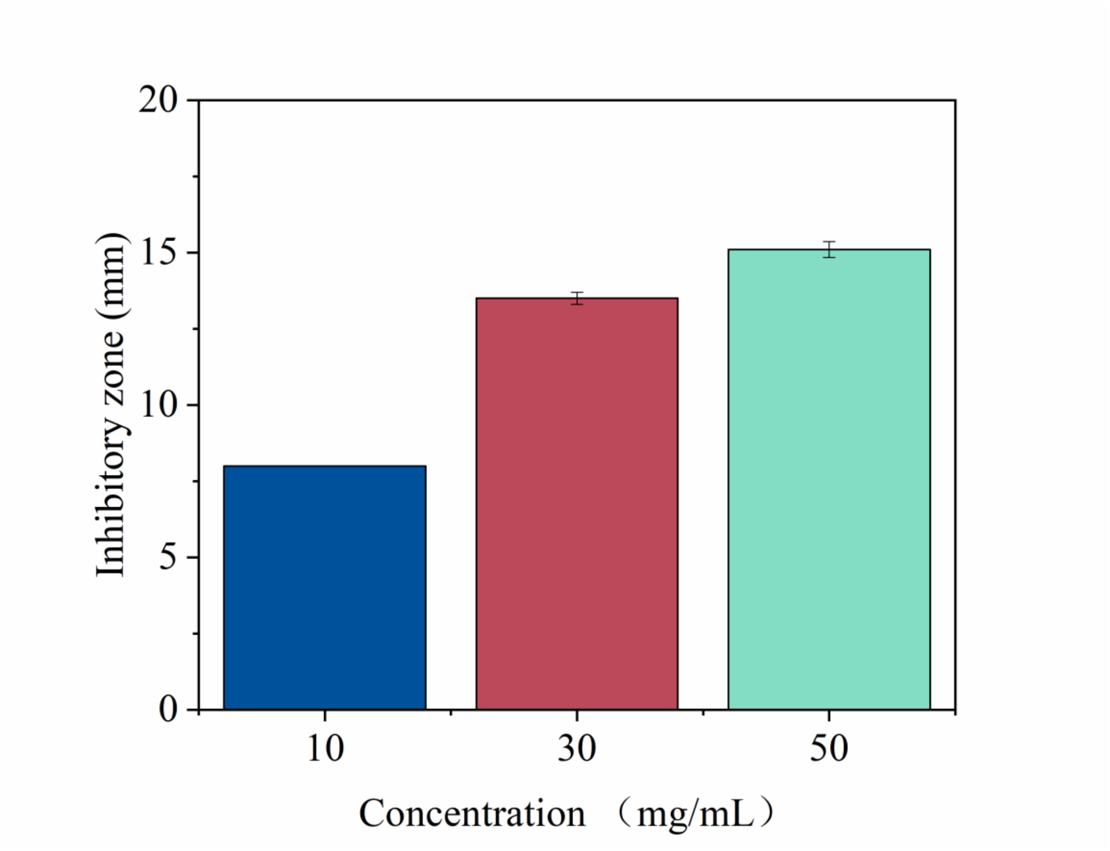

Figure S2. Bacterial growth inhibition zone at different concentrations: A: 10 mg/mL; B: 30 mg/mL; C: 50 mg/mL (n=3).

Table S2. Inhibitory zone of CTS-CHO at different concentrations (n=3).

**Concentration (mg/mL)**	**Average inhibitory zone (mm)**
10	8
30	13.5
50	15.0
